# Genome-scale analysis reveals a role for NdgR in the thiol oxidative stress response in *Streptomyces coelicolor*

**DOI:** 10.1186/s12864-015-1311-0

**Published:** 2015-02-22

**Authors:** Ji-Nu Kim, Yujin Jeong, Ji Sun Yoo, Jung-Hye Roe, Byung-Kwan Cho, Byung-Gee Kim

**Affiliations:** School of Chemical and Biological Engineering, Institute of Molecular Biology and Genetics, and Bioengineering Institute, Seoul National University, Seoul, Korea; Department of Biological Sciences and KAIST institute for the BioCentury, Korea Advanced Institute of Science and Technology, Daejeon, Korea; School of Biological Sciences and Institute of Microbiology, Seoul National University, Seoul, 151-742 Korea

**Keywords:** ChIP-seq, *Streptomyces coelicolor*, NdgR, Transcriptional regulation, Oxidative stress

## Abstract

**Background:**

NdgR is an IclR-type transcription factor that regulates leucine biosynthesis and other metabolic pathways in *Streptomyces coelicolor*. Recent study revealed that NdgR is one of the regulatory targets of SigR, an oxidative stress response sigma factor, suggesting that the NdgR plays an important physiological role in response to environmental stresses. Although the regulatory functions of NdgR were partly characterized, determination of its regulon is required for better understanding of the transcriptional regulatory network related with the oxidative stress response.

**Results:**

We determined genome-wide binding loci of NdgR by using chromatin immunoprecipitation coupled with sequencing (ChIP-seq) and explored its physiological roles. The ChIP-seq profiles revealed 19 direct binding loci with a 15-bp imperfect palindromic motif, including 34 genes in their transcription units. Most genes in branched-chain amino acid and cysteine biosynthesis pathways were involved in the NdgR regulon. We proved that *ndgR* is induced by SigR under the thiol oxidation, and that an *ndgR* mutant strain is sensitive to the thiol oxidizing agent, diamide. Through the expression test of NdgR and the target genes for NdgR under diamide treatment, regulatory motifs were suggested. Interestingly, NdgR constitutes two regulatory motifs, coherent and incoherent feed-forward loops (FFL), in order to control its regulon under the diamide treatment. Using the regulatory motifs, NdgR regulates cysteine biosynthesis in response to thiol oxidative stress, enabling cells to maintain sulfur assimilation with homeostasis under stress conditions.

**Conclusions:**

Our analysis revealed that NdgR is a global transcriptional regulator involved in the regulation of branched-chain amino acids biosynthesis and sulphur assimilation. The identification of the NdgR regulon broadens our knowledge regarding complex regulatory networks governing amino acid biosynthesis in the context of stress responses in *S. coelicolor*.

## Background

Streptomycetes are abundant and ubiquitous soil bacteria that are prolific producers of more than two-thirds of natural antibiotics used in the pharmaceutical industry. They have complex regulatory systems for morphological differentiation in response to environmental or nutritional conditions. Genome sequences of this genus also support the existence of complex regulatory networks for sensing and signal transduction to adapt to such conditions [1-3].

For instance, *S. coelicolor* is the most thoroughly studied model organism among this genus that produces antibiotics, including the red-pigmented tripyrrole undecylprodigiosin (Red), the lipopeptide calcium-dependent antibiotic (CDA), and the deep blue-pigmented polyketide actinorhodin (Act). Its genome size has been revealed as 8,667,507 bp, encoding 965 proteins (12.3%) predicted to have regulatory functions, including sigma factors, two-component regulatory systems, and many transcription factor families such as LysR, LacI, ROK, GntR, TetR, IclR, AraC, AsnC, and MerR [1]. The numbers of its regulatory proteins are much larger than those of *E. coli*, reflecting its differential regulatory networks under changing stress environments.

Members of the IclR family of transcriptional regulatory proteins are composed of approximately 250 amino acid residues [4]. The IclR family has an effector-binding domain at the C-terminus, which is supported by structural data and by studies using mutants [5,6]. The IclR proteins bind their cognate promoters as dimers or as a dimer of dimers with a helix-turn-helix DNA binding motif in the N-terminal domain. The members of this family are known to do repression, activation, or serve as dual-function regulators [4]. Members of the IclR family control a diverse array of genes whose products are involved in glyoxylate shunt in Enterobacteriaceae [7,8], multidrug resistance [9], degradation of aromatics [10-12], inactivation of quorum-sensing signals [13], and determinants of plant pathogenicity and sporulation [14].

In *S. coelicolor*, 16 genes are annotated as members of the IclR family of regulators [4]. Among them, *ndgR* is highly conserved among *Streptomyces* species as well as other actinomycetes such as *Mycobacterium* and *Corynebacterium* [15,16]. NdgR and its orthologs are located adjacent to *leuCD*, which encodes isopropylmalate dehydratase, and have been identified as its transcriptional regulator. In addition, the last step of methionine biosynthesis has been identified as a regulatory target of NdgR [16]. Regulation by *ndgR* orthologs in other bacteria has also been revealed. For example, AreB in *Streptomyces clavuligerus* controls the biosynthesis of leucine and secondary metabolites [17]. In *Corynebacterium glutamicum*, LtbR has been characterized as a regulator involved in leucine and tryptophan biosynthesis [18].

Meanwhile, it was revealed that SigR, an oxidative stress response sigma factor, binds to the upstream region of *ndgR* [19]. SigR is activated via dissociation of the RsrA anti-sigma factor in response to thiol oxidation [20]. This information suggests a physiological role of NdgR as an oxidative stress response regulator.

Previously, we developed a versatile PCR-based tandem epitope tagging tool for the identification of direct binding targets of DNA-binding proteins of *Streptomyces* [21]. Using this tool, we constructed a *S. coelicolor* harboring a 6× myc-tagged NdgR that was successfully utilized for the immunoprecipitation of NdgR-DNA complexes. This experiment revealed that NdgR regulates not only *leuCD* but also most of genes involved in leucine biosynthesis in *S. coelicolor*. Despite the previous studies, the DNA-binding locations of NdgR under physiologically relevant conditions have not been described at a high resolution, and on a genome-wide scale. Here, we further investigate the NdgR regulon *in vivo* using chromatin immunoprecipitation coupled with high-throughput sequencing (ChIP-seq) and further explore the responses of the NdgR regulatory network to thiol oxidative stress. We also discuss the regulatory roles of NdgR in *S. coelicolor*.

## Results and discussion

### Identification of *in vivo* NdgR-binding regions by ChIP-seq

**Figure 1:**
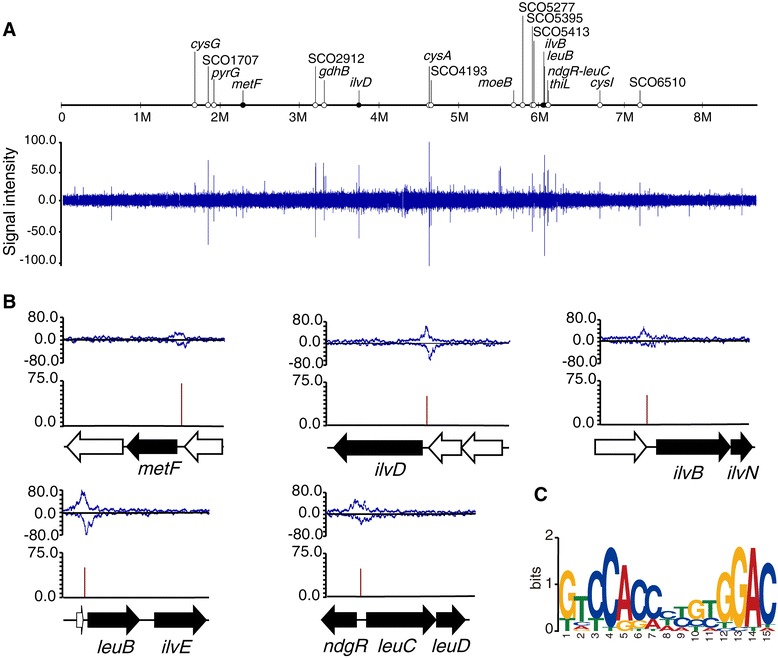
**Genome-wide distributions of NdgR binding regions. (A)** An overview of NdgR binding profiles across the *S. coelicolor* genome when grown on solid minimal media supplemented with N-acetylglucosamine and L-asparagine. Black and white dots indicate previously known and newly found NdgR binding regions, respectively. **(B)** Examples of binding profiles of previously known targets of NdgR. Red lines indicate the locations of putative binding motifs derived from FIMO and the values are the scores for the match of a position. Black arrows indicate the target genes within the transcription units that are directly regulated by NdgR. **(C)** MEME logo representation of the NdgR-DNA binding profile. This motif is present in 18 out of 19 enriched regions identified by ChIP-seq.

NdgR and its orthologs have been characterized *in vitro* by DNA-binding experiments [15-18]. However, *in vivo* analysis of direct interaction between NdgR and its cognate DNA has not been described. To study the binding characteristics of NdgR to the *S. coelicolor* chromosome, we exploited a ChIP-seq method that has been used for genome-wide determination of *in vivo* binding locations of regulatory proteins. We constructed a 6× myc-tagged NdgR strain using homologous recombination for the immunoprecipitation (IP) using a specific anti-c-myc antibody [21]. For cell growth, specific nutrient conditions have often been used to elucidate the function of unknown regulators if the phenotypic differences between wild-type (WT) and deletion mutants were undetectable when the cells were grown in complex media [22]. Thus, we used solid minimal media supplemented with N-acetylglucosamine and L-asparagine for the perturbation of phenotypes [21]. To determine the NdgR-binding regions at the genome scale, we constructed a sequencing library using IP-DNA and performed next-generation sequencing. Sequencing of the library yielded short sequence reads of 36 nucleotides that were uniquely mapped onto the *S. coelicolor* genome (NC_003888). Using the MACS program, 19 NdgR-binding loci were detected with stringent cut-off conditions (*p*-value <1.0e-10, fold enrichment > 3) (Table 1). The peaks were distributed across the entire *S. coelicolor* genome (Figure 1A).

**Table 1:** **Genome-scale identification of NdgR binding regions**

**Peak position**	**Summit**	***p*** **-value** ^**a**^	**Fold enrichment**	**Motif position**	**Consensus sequence**
1662185-1663375	1662816	197.21	4.41	1662781-1662795	GTCCACCCACCGGAC
1827404-1829415	1828601	768.68	6.04	1828423-1828437	GTCCACCACGCGGAC
1896843-1898738	1897467	276.74	5.04	1897508-1897522	GTCCATCCTGCGGAC
2261363-2262539	2261945	101.39	3.54	2261989-2262003	GTCCACCCTTTGGAC
3161458-3164611	3163260	370.89	4.20	3163207-3163221	GATCACACTCCGGAA
3268432-3272122	3269947	139.50	4.06	3269922-3269936	GTTCACCTCGTGGTC
3702919-3706333	3704715	225.72	4.78	3704846-3704860	TTCCACCTTGATCAC
4578730-4584188	4580680	129.09	5.24	4580694-4580708	TCCCACTCCTTGGAC
4600435-4603056	4601769	162.13	4.23	4601788-4601802	GGTCAGCTCCTGGAC
5625694-5627962	5626280	123.46	3.01	N.A.	N.A.
5741825-5743033	5742489	133.14	3.08	5742420-5742434	GACCACCTCGTGGAC
5862424-5864373	5863498	180.16	4.28	5863483-5863497	GTCCACACCGTGGAC
5880856-5884347	5882679	160.34	3.32	5882573-5882587	GTCCGCCTTGAGGAC
6001446-6003903	6002834	279.72	3.77	6002862-6002876	TCCCACCCACTTGAC
6013959-6016845	6015210	329.68	5.54	6015204-6015218	GTCCGCCATGCGGAC
6048531-6053535	6051804	218.57	4.40	6051783-6051797	GTCCAGAACGCCGAC
6059038-6060496	6059807	175.83	4.20	6059774-6059788	GTCCAGCAAGTGGAC
6701783-6704095	6702908	291.94	3.29	6702861-6702875	GTCCACATTTTGGAT
7199604-7200985	7200371	425.57	5.00	7200390-7200404	TTTTGCCATGTGGAC

^a^-10log_10_(*p*-value).

We annotated target genes according to the location of peak summits. If a peak summit was located in ≤ 500 bp upstream or ≤ 100 bp downstream of an annotated start codon, NdgR was considered to regulate the corresponding gene. When the summit was located in an intragenic region between ≥ 100 bp from the start codon of a relevant gene and ≥ 500 bp upstream from the start codon of a downstream gene, this binding state was annotated as an intragenic regulation. Locations of 15 out of 19 peaks were assigned as the intergenic region upstream of transcription units (http://biocyc.org/SCO/organism-summary?object=SCO). Meanwhile, the peak summits of 4 genes (SCO1776, SCO2999, SCO4193, and SCO5277) were located to intragenic regions. With these criteria, we identified 34 genes in the NdgR regulon (Table 2).

**Table 2:** **The NdgR regulon genes**

**SCO no.**	**Name**	**Function**	**Category** ^**a**^	**Note** ^b^
SCO1552		rRNA methylase	2.2.11 RNA synthesis, modification, DNA transcript'n	
SCO1553	*cysG*	Putative uroporphyrin-III methyltransferase	3.2.6 Heme, porphyrin	*
SCO1707		Putative ABC sugar transporter, ATP-binding subunit	1.5.0 Transport/binding proteins	*
SCO1776	*pyrG*	Putative CTP synthetase	3.3.11 Nucleotide interconversions	*, I
SCO2103	*metF*	5,10-methylenetetrahydrofolate reductase	3.1.14 Methionine	*
SCO2910	*cysM*	Cysteine synthase	3.1.6 Cysteine	
SCO2911		Hypothetical protein	0.0.2 Conserved in organism other than Escherichia coli	
SCO2912		Hypothetical protein	0.0.0 Unknown function, no known homologs	*
SCO2999	*gdhB*	Glutamate dehydrogenase	0.0.2 Conserved in organism other than Escherichia coli	*, I
SCO3345	*ilvD*	Dihydroxy acid dehydratase	3.1.21 Valine	*
SCO4164	*cysA*	Putative thiosulfate sulfurtransferase	3.3.19 Sulfur metabolism	*
SCO4165		Hypothetical protein	0.0.2 Conserved in organism other than Escherichia coli	
SCO4193		Putative ATP/GTP-binding membrane protein	4.1.6 Gram + ve membrane	*, I
SCO5178	*moeB*	Putative sulfurylase	3.2.14 Thiamine	*
SCO5277		Magnesium chelatase	7.0.0 Not classified (included putative assignments)	*, I
SCO5395		Putative ABC transporter ATP-binding subunit	1.5.0 Transport/binding proteins	*
SCO5413		Possible MarR-transcriptional regulator	6.3.7 MarR	*
SCO5512	*ilvB*	Acetolactate synthase	3.4.3 Carbon compounds	*
SCO5513	*ilvN*	Acetolactate synthase 3 regulatory subunit	3.1.21 Valine	
SCO5514	*ilvC*	Acetolactate synthase small subunit	3.1.21 Valine	
SCO5522	*leuB*	3-isopropylmalate dehydrogenase	3.1.12 Leucine	*
SCO5523	*ilvE*	Branched-chain amino acid aminotransferase	3.1.21 Valine	
SCO5552	*ndgR*	Putative regulator	6.5.0 Others	*, D
SCO5553	*leuC*	Isopropylmalate isomerase large subunit	3.1.12 Leucine	*, D
SCO5554	*leuD*	Isopropylmalate isomerase small subunit	3.1.12 Leucine	
SCO5562	*thiL*	Thiamin monophosphate kinase	3.2.14 Thiamine	*
SCO5563	*thiD*	Phosphomethylpyrimidine kinase	3.3.14 Thiamine	
SCO6097	*cysN*	Sulfate adenylyltransferase subunit 1	3.3.19 Sulfur metabolism	
SCO6098	*cysD*	Sulfate adenylyltransferase subunit 2	3.3.19 Sulfur metabolism	
SCO6099	*cysC*	Adenylylsulphate kinase	3.3.19 Sulfur metabolism	
SCO6100	*cysH*	Phosphoadenosine phosphosulfate reductase	3.3.19 Sulfur metabolism	
SCO6101		Hypothetical protein	0.0.0 Unknown function, no known homologs	
SCO6102	*cysI*	Putative nitrite/sulfite reductase	3.5.2 Anaerobic respiration	*
SCO6510		Conserved hypothetical protein	0.0.2 Conserved in organism other than Escherichia coli	*

^a^Categories are defined by functional classification of *S. coelicolor* genes in The Sanger Institute database (ftp://ftp.sanger.ac.uk/pub/S_coelicolor/classwise.txt).

^b^Genes with direct binding by NdgR are marked with asterisks (*). Binding of the intragenic regions is denoted as I. Binding of the upstream region between two divergent genes is denoted as D.

Five genes, *metF* (SCO2103), *ilvD* (SCO3345), *ilvB* (SCO5512), *leuB* (SCO5522), and *ndgR*-*leuC* intergenic region (SCO5552-SCO5553) were previously annotated as direct regulatory targets of NdgR by *in vitro* experiments such as electrophoretic mobility shift assay and DNA affinity capture assay [15,16]. The results of the current study show highly enriched profiles of these genes in the ChIP-seq data (Figure 1B). However, *scbR* (SCO6264), which is a gene directly regulated by NdgR [15], was not detected as a target in this experiment. This discrepancy might be due to the differences between *in vivo* and *in vitro* experimental conditions. In addition, we observed low levels of NdgR binding at the promoters of *metH* (SCO1657) and *leuA* (SCO5559) relative to the other binding peaks. Taken together, these results show that binding locations of NdgR were successfully detected *in vivo*.

### Sequence analysis of NdgR-binding regions

Although the putative DNA-binding motifs of NdgR and its orthologs were previously predicted using the known binding sequences of other IclR-type regulators in different strains [15-18], clear consensus sequences remain undefined. To determine the putative NdgR binding motif from our validated ChIP-seq data, 400 nucleotides surrounding the peak summits of 19 binding regions were analyzed by MEME, a bioinformatics tool that identifies overrepresented motifs in multiple unaligned sequences. A 15-bp imperfect palindromic motif ([GT]T[CT]CAC[CA][CTA][TC][GC][TC]GGAC) was detected with an E-value of 1.3e-005 (Figure 1C). This motif was present in 18 out of 19 binding loci, and 13 of them were located within 50 bp of each peak summit. Although the sequence is dissimilar to any known motifs of IclR-type regulators, palindromic sequences of 15 bp have been identified as binding sequences for other IclR-type regulators, and are consistent with a helix-turn-helix interaction [23,24].

We identified the putative binding motif from previously known targets of NdgR as revealed by *in vitro* experiments such as electrophoretic mobility shift assay and DNA affinity capture assay [15,16]. This motif was detected in upstream regions of *metF*, *ilvB*, *ilvD*, *leuB*, and the intergenic region between *NdgR* and *leuCD*. The putative motif from genome-wide prediction using FIMO was found in the promoters of known targets, *metH* and *leuA*. This result further supports that their low intensity in ChIP-seq profiles represents the subtle differences between *in vivo* and *in vitro* experimental conditions.

### Functional classification of the NdgR regulon

**Figure 2:**
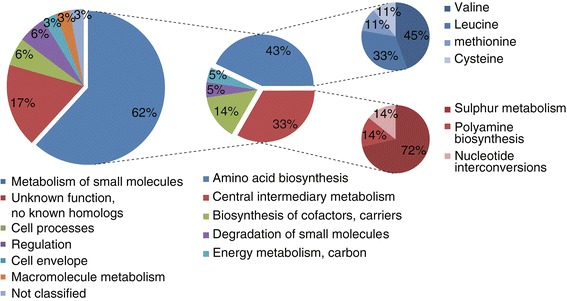
**Functional classification of genes in the NdgR regulon.** Hierarchical functional class is defined by The Sanger Institute database. Genes in this chart are described in Table 2.

Genes in the NdgR regulon were further classified into functional categories according to gene classifications defined by The Sanger Institute database (ftp://ftp.sanger.ac.uk/pub/S_coelicolor/classwise.txt) (Figure 2). The metabolism of small molecule category is highly dominant (62%, 21/34 genes). Among the genes in this category, 43% and 33% were assigned to amino acid biosynthesis (9/21 genes) and central intermediary metabolism (7/21 genes), respectively, and 14% (3/21 genes) were included in biosynthesis of cofactors and carriers.

**Figure 3:**
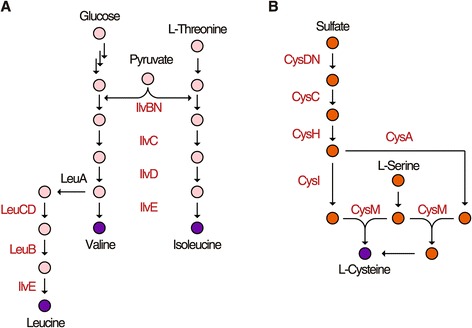
**Metabolic pathways directly regulated by NdgR.** The proteins identified by ChIP-seq are depicted by red characters. **(A)** NdgR directly regulates genes in most steps of the BCAA biosynthesis pathways. Though LeuA was not annotated as a member of NdgR regulon, the putative motif from genome-wide prediction using FIMO and low binding signal in ChIP-seq data was observed in its upstream region. **(B)** The sulfur assimilation into the cysteine biosynthesis pathways.

NdgR directly regulates eight genes in the biosynthetic pathways of branched chain amino acids (BCAAs) (Figure 3A). For instance, the first step in BCAA biosynthesis is catalyzed by acetohydroxy acid synthase/acetolactate synthase encoded by *ilvBN*. This enzyme catalyzes the condensation of two pyruvate molecules to acetolactate and 2-acetohydroxybutyrate from pyruvate and 2-ketobutyrate. The following reaction is catalyzed by ketol-acid reductoisomerase and dihydroxy-acid dehydratase encoded by *ilvC* and *ilvD*, respectively. The final transamination step, as well as the first step in the degradation pathways, is catalyzed by BCAA aminotransferases encoded by *ilvE*. Leucine is synthesized from α-ketoisovalerate, an intermediate in the valine pathway, through three enzymatic steps. The relevant enzymes are α-isopropylmalate synthase (LeuA), β-isopropylmalate dehydratase (LeuC, LeuD), and β-isopropylmalate dehydrogenase (LeuB). Despite the scattered locations of those genes along the chromosome, NdgR directly bound their upstream regions. Interestingly, the NdgR mutant (BG11) exhibited methionine auxotrophy, but not leucine auxotrophy [16]. Notwithstanding its direct binding at the promoters of genes in the BCAA biosynthetic pathway, NdgR was not essential for BCAA biosynthesis under the growth conditions used here. Thus, it is expected that NdgR plays a role in the fine-tuning of BCAA biosynthesis with the assistance of feedback regulation and translational attenuation common in amino acid biosynthetic pathways in bacteria [25-27].

Next, we observed that NdgR directly bound the upstream region of three transcription units including seven genes (*cysA*, *cysM, cysN*, *cysD*, *cysC*, *cysH* and *cysI*) that are involved in sulfur-assimilation metabolism (Figure 3B). The pathway of sulfur assimilation into cysteine biosynthesis in *S. coelicolor* has been suggested in previous reports [28,29]. The genes, including *cysN* (SCO6097), *cysD* (SCO6098), and *cysC* (SCO6099), participate in 3′-phosphoadenylyl sulfate (PAPS) formation from sulfate. PAPS reductase encoded by *cysH* (SCO6100) converts PAPS to sulfite. The serial reactions are followed by two pathways that result in thiosulfate production by a thiosulfate sulfurtransferase and sulfide production by a sulfite reductase encoded by *cysA* (SCO4164) and *cysI* (SCO6102), respectively. The two metabolites (thiosulfate and sulfide) are sulfur donors for the sulfur assimilation into O-acetyl-L-serine by cysteine synthase encoded by *cysM* (SCO2910) [30,31].

Moreover, NdgR bound to the promoter region of putative siroheme synthase encoded by *cysG* (SCO1553). Siroheme is a prosthetic group that participates in six-electron reduction reactions catalyzed by both sulfite and nitrite reductases. CysG converts uroporphyrinogen III, which is a precursor of heme and cobalamin (vitamin B12), to siroheme using multifunctional activities such as SAM-dependent methylase, dehydrogenase and ferrochelatase [32]. Biosynthesis of sulfur-containing thiamine was also regulated by NdgR. As the active form of thiamine, thiamine pyrophosphate (TPP) is an essential cofactor for particular metabolic processes such as BCAA biosynthesis. The thiamine category includes thiamine monophosphate kinase and sulfurylase encoded by *thiL* (SCO5562) and *moeB* (SCO5178), respectively. In addition, phosphomethylpyrimidine kinase encoded by *thiD* (SCO5563) in the polyamine biosynthesis category is likely involved in thiamine metabolism.

In *S. clavuligerus*, AreB, an ortholog of NdgR, bound to the upstream region of the pathway-specific regulator of clavulanic acid and cephamycin C, and the *areB* deletion mutant increased their production. Moreover, the *ndgR* mutant showed overproduction of actinorhodin in our experimental conditions [21]. Interestingly, though some effects of secondary metabolites have been observed in the mutants of *ndgR* and its ortholog [15,17], none of the secondary metabolite genes were detected as NdgR targets. This observation indicates that NdgR indirectly regulates the genes involved in secondary metabolism. Taken together, the data suggest that NdgR mainly regulates primary metabolism of small molecules, especially BCAA and several sulfur-containing molecules.

### Role of NdgR under thiol oxidative stress

**Figure 4:**
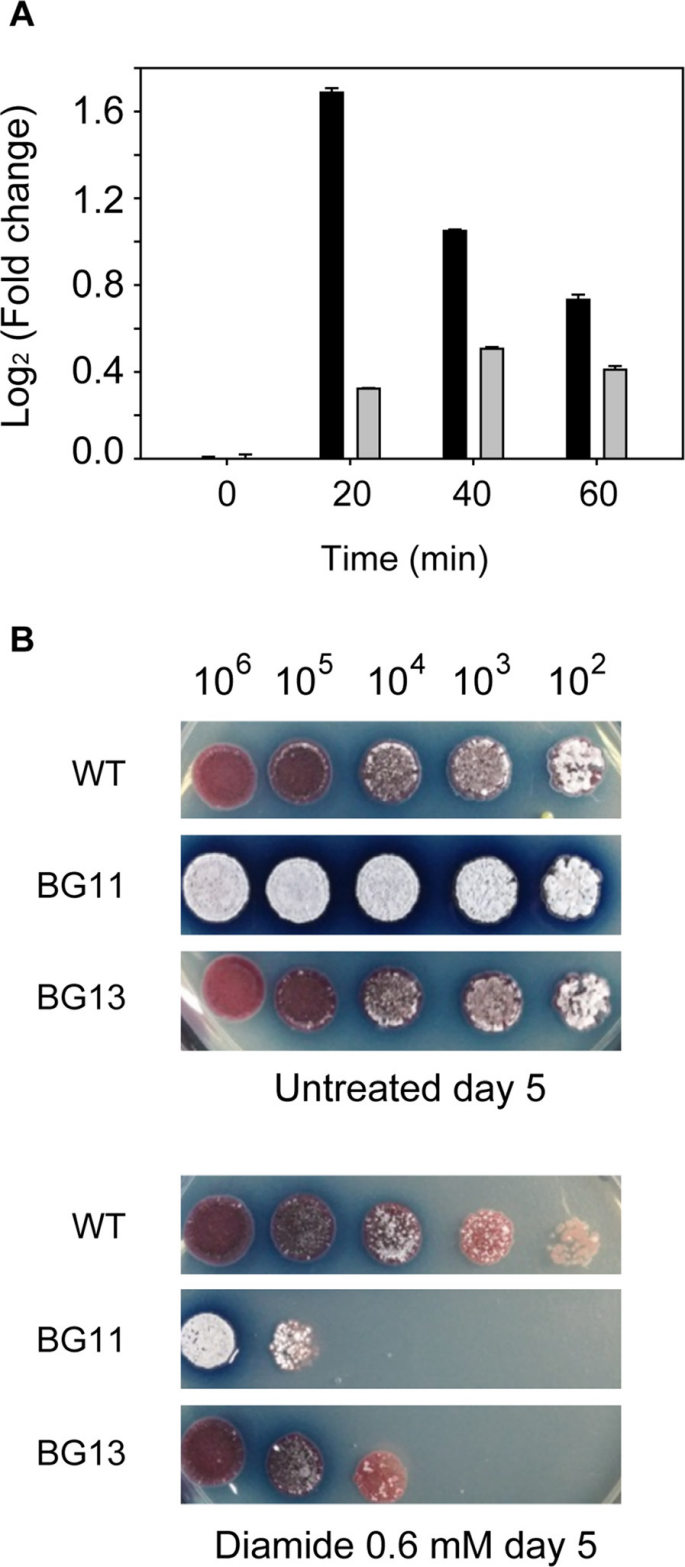
**SigR-dependent transcription activation of the** ***ndgR*** **gene in** ***S. coelicolor.*** **(A)** Quantitation of *ndgR* transcripts using quantitative realtime-PCR analysis (qRT-PCR) from diamide-treated cells reveals that the *ndgR* gene is induced using the SigR promoter. mRNA level of *ndgR* in WT (black bars) was induced under diamide treatment compared to the level of *ndgR* in *sigRrsrA* deletion mutant (gray bars). All levels are normalized by the levels of each sample at 0 min. **(B)** Sensitivity test of WT, *ndgR* deletion mutant (BG11) and complemented mutant (BG13) under thiol oxidative stress. Serially diluted spores of WT, BG11 and BG13 were spotted on R5 agar plates with or without added diamide (0.6 mM). Plates were incubated at 30°C for 5 days.

To ascertain the physiological role of the IclR family of regulators in the cell, identification of the interaction between the ligand and the substrate-recognition domain of the regulator would provide valuable insight. The effector molecule of NdgR has not been identified despite several attempts [15,16]. Instead, we explored a higher level of the NdgR regulatory network. Previous ChIP-chip experiments revealed *ndgR* as one of the targets of the oxidative stress response sigma factor SigR [19]. We measured mRNA expression level to validate the transcription of *ndgR* by SigR in response to thiol oxidative stress. NdgR was transiently induced by diamide treatment in the presence of SigR (Figure 4A); hence, NdgR is expected to regulate its target genes in response to thiol oxidative stress. However, NdgR was expressed constitutively in the absence of SigR, suggesting that there are additional transcriptional regulators in addition to the SigR.

Next, we sought to examine whether the *ndgR* mutant strain is sensitive to this thiol-reactive compound using a plate assay. We used complex media for the sensitivity test because *ndgR* mutant hardly grows in minimal media [15,16]. When WT, *ndgR* mutant (BG11) and complemented mutant (BG13) spores were spotted on the R5- agar plates containing 0.6 mM diamide, BG11 cells were found to be sensitive to diamide even in the complex media (Figure 4B). These results show that *ndgR* is necessary for the response to oxidative stress conditions.

The role of BCAAs in response to stress conditions has not been completely elucidated in bacteria; however, *leuCD* induction in response to thiol-specific oxidative stress was reported in *Mycobacterium bovis* BCG [33]. In plants, there have been many reports regarding the accumulation of BCAAs in abiotic stress conditions [34-36]. It has been suggested that BCAAs function as compatible osmolytes since the level of BCAAs is elevated under drought stress in various plant tissues [36]. Another possible role for BCAAs under stress conditions that has been suggested is that they function as alternative electron donors for the mitochondrial electron transport chain via the electron transfer flavoprotein (ETF) complex to produce ATP [35]. Isovaleryl-CoA from the degradation of BCAAs provides electrons to the ETF complex via the action of isovaleryl-CoA dehydrogenase. In addition, we observed that the *ndgR* mutant exhibited defective membrane formation in minimal media (data not shown). This result is likely due to the fact that 70% of total fatty acids in the membrane are branched-chain fatty acids, which are synthesized from the precursors derived from BCAA degradation [37].

Sulfur-related reactions are well known as anti-oxidation reactions in actinomycetes [19,20,28]. Expression levels of *cysIHCDN*, *cysM*, and *cysA* are significantly increased by SigB under osmotic and oxidative stresses [28], leading to an increase in cysteine levels. As a component of mycothiol, a major thiol buffer found in many actinomycetes, cysteine would protect the cell against osmotic and oxidative stresses. Because oxidation-labile S-containing factors such as TPP and iron-sulfur clusters are involved in many physiological reactions, NdgR would contribute to replenishing these factors. Thus, we speculate that NdgR maintains the intracellular redox balance and the structural integrity of the membrane in response to external thiol oxidative stress by orchestrating the genes in its regulon.

### Elucidation of NdgR regulatory logic

**Figure 5:**
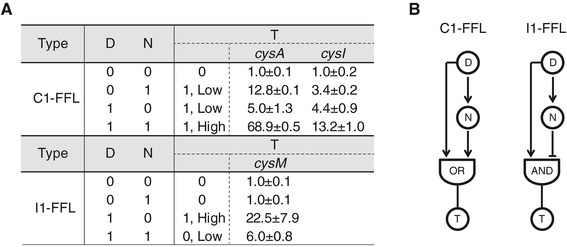
**The regulatory modes of NdgR. (A)** Measurement of expression levels of NdgR target genes in the sulfate assimilation pathway in various combinations of input signals. D, N and T denote diamide treatment, *ndgR* gene and target genes of NdgR, respectively. D = 0 or 1 indicates nontreatment or treatment of diamide, respectively. The absence or presence of the *ndgR* gene is denoted as 0 or 1, respectively. Expression of target genes above threshold are denoted as 0 (OFF) or 1 (ON) of output signals. Expression levels were normalized relative to the expression levels of controls (D = 0, N = 0). All the values are represented with standard deviations. **(B)** The logic gates of NdgR regulatory networks. NdgR regulates the sulfate assimilation pathway using coherent and incoherent FFL.

The members of the IclR family of regulators have been demonstrated to be activators, repressors, and dual-role proteins in many cases [4]. However, to ascertain the physiological role of a regulator, a comprehensive understanding of its regulatory modes, including higher and lower levels of regulation, would be helpful. In order to elucidate how NdgR regulates target gene expression in response to oxidative stress, we quantified mRNA levels of the relevant genes using qRT-PCR (Figure 5A). We selected sulfur assimilation into the cysteine biosynthesis pathway as a target due to its significance in the stress response [28]. The selected genes were *cysI* in *cysIHCDN* operon, *cysA* and *cysM* in cysteine biosynthesis, and *ndgR*. First, we confirmed that the transcriptional level of *ndgR* is induced by diamide. All of the target genes were also induced by diamide regardless of the presence of *ndgR*. This result indicates that other transcription factors related to oxidative stress could also exist for the regulation of them. Next, we observed two regulatory modes based on the measurement of expression levels affected by NdgR under diamide treatment. *cysA* and *cysI* were induced by NdgR regardless of diamide. Meanwhile, *cysM* seems to be hardly expressed without diamide treatment regardless of the presence of *ndgR*. Although NdgR may bind to the promoter of *cysM* in absence of diamide treatment, the expression level may not be decreased because *cysM* is not expressed originally. However, NdgR repressed the expression of *cysM* in the presence of diamide; thus, the role of NdgR as a dual regulator was confirmed.

We further explored the physiological roles of these two regulatory modes by NdgR using network motif theory [38]. The two regulatory modes of NdgR were coherent type-1 feed-forward loop (C1-FFL) with OR-gate, and incoherent type-1 feed-forward loop (I1-FFL) (Figure 5B). A C1-FFL is a regulatory pattern in which an activator (D) controls a target gene (T) and also activates another activator (N) of that target gene. C1-FFL with OR-gate shows a delayed response to OFF steps of D and a rapid response to ON steps [38]. Because sulfur assimilation governed by *cysI* and *cysA* is controlled by C1-FFL with OR-gates, cells can maintain sulfur assimilation activity after the signal is OFF. Meanwhile, an I1-FFL is a regulatory pattern in which an activator (D) controls a target gene (T) and also activates a repressor (N) of that target gene. This motif is a pulse generator and a response accelerator [38]. In addition, I1-FFL provides fold-change detection that responds only to the fold-change (rather than absolute change) of the input signal [39]. CysM has an I1-FFL network motif; thus, it may be expressed in a rapid response that is proportional to the fold-change in the stimulus relative to the background.

We revealed that NdgR controls sulfur assimilation into the cysteine biosynthesis pathway through two regulatory modes. Using C1-FFL with OR-gate, NdgR can initiate a reduction in sulfate immediately in response to the stress and stably protect assimilation systems against transient loss of signal. At the final step, NdgR acts as a memory of stress intensity by using the I1-FFL motif. Thus, it mediates a continual temporal comparison between the present and past levels of stresses. During the stress condition, the memory is adjusted to the new level of stress intensity, and cysteine synthesis by CysM returns to its basal level. Thus, this modulation prevents excess synthesis of cysteine, which is energetically wasteful and avoids potential osmotic imbalances. Using these regulatory modes, NdgR likely provides an advantage for *S. coelicolor* to maintain homeostasis in stress conditions.

## Conclusions

We identified genome-wide binding sites of NdgR in *S. coelicolor* using ChIP-seq and revealed its physiological role under oxidative stress conditions. In our growth condition, we found that NdgR directly regulates 34 genes that are involved in the synthesis of BCAAs and cysteine using 19 regulatory binding sites. We confirmed that SigR, an oxidative stress response sigma factor, induces NdgR in response to thiol oxidative stress induced by diamide treatment. Interestingly, this implied physiological roles of the NdgR regulon in *S. coelicolor*. Degradation of BCAAs is known to produce major CoA precursors of branched-chain fatty acids, which are the major components of bacterial cell membranes; thus, their production enhances the robustness of the cell. Furthermore, BCAAs can serve as an alternative electron transport donor for energy production under various stress conditions, similarly to that which occurs in plants. Because the induction of biosynthesis of BCAA under stress conditions has also been reported in other bacteria, the exact roles of BCAAs in stress conditions require further investigation. In addition, many BCAA biosynthesis genes require sulfur-containing cofactors such as thiamine and iron-sulfur clusters, which are vulnerable to thiol oxidative stress; these cofactors can be replenished by sulfur-related pathways such as cysteine biosynthesis, which is also regulated by NdgR. Furthermore, cysteine is one of the precursors of mycothiol, a major redox buffer in *S. coelicolor* that helps maintain redox balance in the cell. The cysteine biosynthesis pathway is regulated by NdgR under thiol oxidative stress using coherent and incoherent FFL, which enables cells to adapt to the environmental conditions by maintaining homeostasis. This study provides a deeper understanding the mechanisms by which NdgR regulates amino acid biosynthesis in response to stress in *S. coelicolor*. In addition, this model system shows a possibility such that revealing the global regulatory network of transcription factors using ChIP-seq technique will enable in depth understanding of their physiological roles in *S. coelicolor*.

## Methods

### Bacterial strains and growth conditions

All strains used are *S. coelicolor* A3 (2) M145 and *E. coli* K-12 MG1655 and its derivatives. For the ChIP experiments, strain expressing 6× myc-tagged NdgR constructed previously [21] was grown on solid minimal media composed of 0.05% (w/w) K_2_HPO_4_, 0.02% MgSO_4_ · 7H_2_O, 0.001% FeSO_4_ · 7H_2_O, 2.2% agar, 0.05% L-asparagine, and 1% N-acetylglucosamine. For sensitivity testing and gene expression analysis, spores of *S. coelicolor* A3(2) M145 and an *ndgR* knockout strain, previously constructed using PCR targeting method [15,16,40], were grown in R5- medium composed of 10.3% sucrose, 0.025% K_2_SO_4_, 1.01% MgCl_2_ · 6H_2_O, 1% glucose, 0.01% Difco casamino acids, 0.2% trace element solution composed of 0.004% ZnCl_2_, 0.02% FeCl_3_ · 6H_2_O, 0.001% CuCl_2_ · 2H_2_O, 0.001% MnCl_2_ · 4H_2_O, 0.001% mg Na_2_B_4_O_7_ · 10H_2_O, 0.001% mg (NH_4_)_6_Mo_7_O_24_ · 4H_2_O in 1 l of deionized water, 0.5% yeast extract, 0.57% TES buffer, and 0.7% (v/v) 1 N NaOH in 1 l of distilled water.

### ChIP-seq analysis

ChIP experiments were performed as described previously [21]. Cells harboring 6× myc-tagged NdgR were grown in solid minimal media supplemented with N-acetylglucosamine and asparagine at 30°C for 36 hr. The cells were then cross-linked with 1% formaldehyde at room temperature for 30 min. Following the quenching of unused formaldehyde with 125 mM glycine at room temperature for 5 min, the cross-linked cells were harvested by centrifugation and washed three times with 50 ml ice-cold Tris-buffered saline (Sigma-Aldrich, St. Louis, MO, USA). The washed cells were resuspended in 1.5 ml lysis buffer composed of 10 mM Tris–HCl (pH 7.5), 100 mM NaCl, 1 mM EDTA, protease inhibitor cocktail (Sigma-Aldrich), and 1 kU lysozyme (Epicentre, Madison, WI, USA). The cells were incubated at room temperature for 30 min and then treated with 2 ml of 2× IP buffer (100 mM Tris–HCl, pH 7.5, 200 mM NaCl, 1 mM EDTA, 2% Triton X-100). The lysates were then sonicated eight times for 20 s each in an ice bath to fragment the chromatin. The DNA size range resulting from the sonication was 300–1000 bp, and the average DNA size was 500 bp. Cell debris was removed by centrifugation at 37,000 × g for 10 min at 4°C, and the resulting supernatant was used as the cell extract for immunoprecipitation. To immunoprecipitate the NdgR–DNA complex, 3 μg of anti-c-myc antibody (9E10, Santa Cruz Biotech, Santa cruz, CA, USA) was added to the cell extract. For the nonspecific control (mock-IP), 2 μg of normal mouse IgG (Millipore, Billerica, MA, USA) was added to the cell extract. The samples were then incubated overnight at 4°C, followed by the addition of 50 μl of Dynabeads Pan Mouse IgG beads (Invitrogen, Carlsbad, CA, USA). After 5 h of incubation at 4°C, the beads were washed twice with IP buffer (50 mM Tris–HCl at pH 7.5, 140 mM NaCl, 1 mM EDTA, 1% (v/v) Triton X-100), once with wash buffer I (50 mM Tris–HCl at pH 7.5, 500 mM NaCl, 1% (v/v) Triton X-100, 1 mM EDTA), once with wash buffer II (10 mM Tris–HCl buffer at pH 8.0, 250 mM LiCl, 1% (v/v) Triton X-100, 1 mM EDTA), and once with TE buffer (10 mM Tris–HCl at pH 8.0, 1 mM EDTA). After removing the TE buffer, the beads were resuspended in 200 μl of elution buffer (50 mM Tris–HCl at pH 8.0, 10 mM EDTA, and 1% SDS) and incubated overnight at 65°C. RNA was then digested by incubation with 200 μl of TE buffer and 1 μl RNaseA (Qiagen, Hilden, Germany) for 2 h at 37°C. Proteins in the DNA sample were then digested by incubation with 4 μl of proteinase K solution (Invitrogen) for 2 h at 55°C. The sample was then purified with a PCR purification kit (Macherey-Nagel, Dueren, Germany). Next-generation sequencing was carried out using an Illumina Genome Analyzer IIx (Illumina, San Diego, CA, USA) with single reads of 36 bp each.

### Sequence analysis

Illumina reads were aligned to the *S. coelicolor* reference genome (GenBank: NC_003888) with CLC Genomics Workbench 6.5. The alignment BAM file was analyzed using MACS v1.4 to detect read-enriched regions in the genome [41]. Enriched regions were selected for further study based on scores greater than 100 using -10Log_10_ (*p*-value), and fold-enrichment greater than 3. For conserved motif searches, nucleotide sequences (400 bp) centered on each peak were extracted and submitted to the MEME software suite [42]. The parameters used were maximum length of 20 nucleotides and zero or one per sequence of each submitted sequence. The FIMO program in the MEME suite was used for searching genome-wide occurrences of the putative motifs derived from MEME.

### Sensitivity test

The *ndgR* disruption mutant (BG11) and *ndgR* complemented mutant (BG13) were obtained using a PCR-targeting mutagenesis protocol as described previously [15,16,40]. For diamide sensitivity assay, the wild-type, BG11 and BG13 spores (10^6^, 10^5^, 10^4^, 10^3^, and 10^2^) were spotted on R5- agar containing 0.6 mM diamide or 0 mM diamide (control) and incubated for 5 days at 30°C.

### Gene expression analysis

Total RNA extraction was performed with the RNeasy Mini Kit (Qiagen) according to the manufacturer’s instructions. Sample aliquots were obtained after 30 min of incubation with or without 0.6 mM diamide at the exponential phase (OD600 ~ 0.5). RNA was reverse transcribed into first-strand cDNA using SuperScript™ III Reverse Transcriptase (Invitrogen). qPCR was performed using a CFX96 real-time system (Bio-rad, Hercules, CA, USA). All samples were measured in triplicate. Gene expression level was normalized by using housekeeping gene (SCO5820) as internal control.

### Availability of supporting data

All raw sequence data files have been deposited to Gene Expression Omnibus through accession number GSE59010.

## Abbreviations

FFL: Feed-forward loop
ChIP-seq: Chromatin immunoprecipitation coupled with sequencing
WT: Wild-type
BCAA: Branched-chain amino acid
TPP: Thiamine pyrophosphate
ETF: Electron transfer flavoprotein
C1-FFL: Coherent type-1 feed-forward loop
I1-FFL: Incoherent type-1 feed-forward loop
